# Antidiabetic effects of Peganum harmala seed extract on streptozotocin-induced diabetes in rats

**DOI:** 10.22038/ajp.2024.25241

**Published:** 2025

**Authors:** Mozafar Khazaei, Mohammadali Meskaraf-asadabadi, Elham Ghanbari, Amir Hossein Khazaei

**Affiliations:** 1 *Fertility and Infertility Research Center, Health Technology Research Institute, Kermanshah University of Medical Sciences, Kermanshah, Iran *; 2 *Department of Tissue Engineering, School of Medicine, Kermanshah University of Medical Sciences, Kermanshah, Iran *; 3 *Student Research Committee, Shahid Beheshti University of Medical Sciences, Tehran, Iran *

**Keywords:** Diabetes, Pancreas, Beta Cell, Streptozotocin, Peganum harmala, Regeneration

## Abstract

**Objective:**

Diabetes, a chronic metabolic disease, has many complex complications and an increasing prevalence in various societies. Despite conventional drug treatments and limited surgical and tissue transplant methods, a definitive diabetes treatment remains to be found. Restoring damaged beta cells to insulin production or prompting other pancreatic cells to secrete insulin is an essential goal of diabetes research. The present study investigated the antidiabetic and regenerative effects of *Peganum harmala* seed extract (*PHSE*) on streptozotocin (STZ)-induced diabetes in rats.

**Materials and Methods:**

In this experimental *in vivo* study, male Wistar rats (200±10 g) were placed in 5 groups: control, untreated diabetic and diabetic groups treated with 100, 200, and 400 mg/kg doses of *PHSE*. Fasting blood sugar (FBS), C-peptide, insulin, and antioxidant parameters (total antioxidant capacity (TAC) and nitric oxide (NO)) of serum were measured. Pancreatic tissue was used for histologic staining and assessment of the expression of genes related to beta cell regeneration.

**Results:**

*PHSE* significantly improved FBS, weight loss, insulin, c-peptide, TAC, NO, and expression of pancreatic genes (*insulin, PDX1 *and *neurogenin-3*) (p<0.05). It also increased the number of pancreatic beta cells.

**Conclusion:**

*PHSE* has considerable regenerative and antidiabetic effects on changes caused by diabetes in rats’ serum and pancreas.

## Introduction

Diabetes is the most common metabolic disease and a main cause of concern in human societies with its exponential prevalence, which has increased from 8.3% (366 million people) in 2011 to approximately 10% (522 million people) in 2030 (Williams et al. 2020). Diabetes is a complex disease with side effects, and its financial costs are very high. The financial burden related to the care of this disease is currently about $760 billion USD and is expected to reach $825 billion USD in the next ten years (Luo et al. 2021).

The two main types of diabetes are type 1 (T1D), an autoimmune disease, and type 2 (T2D), caused mainly by lifestyle. The disease affects a slightly higher percentage of men (9.8%) than women (9.2%). More than 1.1 million children and adolescents under the age of 20 suffer from T1D, and this number increases every year (James et al. 2023). This disease is caused by the invasion of T lymphocytes and macrophages into pancreatic islets, which leads to the loss of beta cells (Roep et al. 2021).

When the symptoms of T1D appear, more than 70% of beta cells have already been destroyed. The remaining beta cells, however, may serve as an important source for beta-cell renewal (Oram et al. 2019). Studies have shown that in T1D, the presence of autoantibodies against the isoform of glutamic acid decarboxylase, insulinoma-associated protein 2 (IA2) or islet cell antigen (ICA), and zinc transporter 8 (ZnT8) as well as changes in the capacity of regulatory T cells suppress the activity of effector T cells (Teff cells), which play a prominent role in destructive processes. Some studies have focused on suppressing the immune system to prevent and cure T1D (Khazaei et al. 2023; Roep et al. 2021); yet such treatment methods have shown little hope, and many of them have had reverse and negative results. 

Therefore, identifying new therapeutic agents particularly edible natural oral compounds that can preserve, re-establish, and restore beta cell function, can be of great clinical use. Insulin injection is the most essential and available treatment for T1D; however, many patients show not only poor metabolic control, but also hemoglobin A1C levels that remain above seven as well as further complications such as hypoglycemia, overweight, and dyslipidemia. Additionally, reduced glucose absorption by skeletal muscles and insulin resistance can be seen, which in turn, causes chronic complications in T1D (Wolosowicz et al. 2020). 

Oxidative stress plays an essential role in the onset, progression, and chronic complications of T1D. Hyperglycemia caused by oxygen free radicals leads to decreased insulin secretion, affecting its sensitivity and signaling in tissue response (González et al. 2023). Apoptosis caused by oxidative stress is another mechanism involved in the destruction of pancreatic beta cells. Maintaining and improving the remaining capacity for insulin production and reducing pancreatic inflammation in patients with T1D are particularly important treatment goals (Dinić et al. 2022). Various studies on T1D and T2D have reported that the oral use of particular compounds helps to improve insulin resistance and secretion and reduce hyperglycemia and its complications. 

To date, more than 1200 medicinal plants that have positive effects on reducing fasting blood sugar (FBS) levels or the complications caused by diabetes have been identified, and many of them have been the subject of recent laboratory and clinical research studies into their use to treat diabetes. Some such studies have obtained significant results in reducing FBS of diabetic patients (Khazaei et al. 2018b; Kifle et al. 2022). 

Traditional medicine has long recommended the use of *Peganum harmala* L. (Zygophyllaceae family) as a sedative, a hypnotic, an anthelmintic, a diuretic, and an aphrodisiac (Li 2024). *P. harmala* is an herbaceous plant with a wide distribution and it contains compounds such as beta-carboline alkaloids, the most famous of which are harmine, harmaline, and harmalol (Iranshahy et al. 2019). Harmaline however, strengthens the central nervous system and is toxic in high doses. Immoderate consumption of *P. harmala* seed *(PHS) *is poisonous, and therefore, its oral utilization is done with caution (Asadzadeh et al. 2021). 

A few exciting studies in recent years have reported the effects of *PHS* and its compounds, such as harmine, on diabetes. In an animal study, a study was reported the antidiabetic effects of *PHS* extract *(PHSE)* on FBS, HbA1c, and liver enzymes in an streptozotocin (STZ)-induced T1D model (Komeili et al. 2016). Another study reported the anti-apoptotic effects of methanol extract of *PHS* on STZ-induced diabetes in adult male rats (Fahimi et al. 2022). Researchers monitoring more than 100,000 possible compounds for beta cell regeneration found that harmine improved human insulin-producing beta cell development and may be considered a treatment for T1D (Lotfi and Khazaei 2021).

Considering the accessibility of herbal sources, their fewer side effects, the acceptability of herbal therapy by most patients, and the lack of restrictions on the production or importation of medicinal raw materials as well as the good results of previous research into an effective herbal therapeutic source for diabetes, further research seems necessary. The current study aimed to investigate the effects of *PHSE* on serum and tissue changes caused by T1D induced in rats.

## Materials and Methods

### Study design

In this experimental *in vivo* study, male Wistar rats (200±10 g) were divided into five groups (n=5): control, untreated diabetic and diabetic treated with 100, 200, and 400 mg/kg doses of *PHSE *orally once a day for 21 days. Animals were weighed on days 7, 14, and 21, and FBS was measured using a Bionem glucometer (Taichung City, Taiwan). Diabetes was induced in fasting rats by intraperitoneal (i.p.) injection of a single dose of STZ (55 mg/kg) dissolved in citrate buffer (Lotfi and Khazaei 2021). *PHS *was prepared from local market and confirm by herbal Dep. of pharmacy school (voucher number. *Pegnum harmala*-Esfand: KUMS-403PMP), and its hydroalcoholic extract was prepared using 70% ethanol in percolation method (Khazaei et al. 2018b). Amounts and duration of treatment were determined based on previous studies on the plant and similar studies (Asadzadeh et al. 2021; Komeili et al. 2016; Li 2024) on treating induced diabetes with plant extracts and IC50 (i.p and oral doses of different extracts). To determine toxic dose (lethal dose (LD) 50) of* P. harmala*, i.p injection of *PHSE* in small mice was done. 

After the 21-day treatment period, FBS of all animals were determined and two additional rats of each treated group were kept for one month to find changes of FBS levels. The animals were euthanized with ketamine 10% (15 mg/kg, i.p.) and xylazine 2% (80 mg/kg i.p.) and heart blood samples were taken; serum was separated and kept at -20°C to measure hormonal and biochemical markers. Immediately, pancreatic tissue was dissected, and a part of each pancreas was fixed in formalin to prepare tissue sections for hematoxylin and eosin (H&E) and modified aldehyde fuchsin (A.F) staining. Another part was placed in liquid nitrogen to examine the expression of genes related to the survival and differentiation of beta cells. FBS, C-peptide, insulin, nitric oxide (NO), and total antioxidant capacity (TAC) were determined by Griess assay and the ferric reducing antioxidant power (FRAP) method.

### Measurement of serum TAC and NO

C-peptide and insulin were measured using specific Elisa kits for rats (SunLong Biotech, China), and antioxidant indices (TAC and NO) were determined using special protocols. The TAC of serum samples was measured using the FRAP method and the reduction of Fe^+3^ ions to Fe^+2 ^in the presence of tripyridyltriazine at a wavelength of 593 nm by spectro- photometry (Khazaei et al. 2018a). NO values were measured using Griess's colorimetric method. Since direct NO measurement is challenging, the amounts of nitrite and nitrate are measured as its indicators. To this end, serum samples were deproteinized using zinc sulfate and centrifugation. Then, sulfanilamide (2%) was added to 0.1% ethylene diamide dihydrochloride and incubated for 30 min at 37°C. Finally, the obtained color was read at wavelengths of 540 and 630 nm, the absorbance of the samples was compared with the absorbance standard (0 to 200 micromolar sodium nitrate), and the NO concentration was calculated (Khazaei et al. 2019). 

### Tissue staining

After processing the fixed tissue samples, paraffin embedding, and sectioning, serial 5-µm sections were prepared and used alternately with the H&E staining method for general evaluation of cell and tissue, and modified A.F method which is a specific histological staining protocol for pancreatic bet cell conformation (Bancroft and Gamble 2008). Multiple images were taken of the sections of each sample at magnifications of 40, 100, and 400 using a Motic microscope and camera (Moticam 2000, Spain), and tissue changes were recorded from comparisons among the groups. 

### Real-time polymerase chain reaction (Real-time PCR)

Real-time polymerase chain reaction (Real-Time PCR) was used to evaluate the expression of *insulin, pancreatic duodenal homeobox 1 *(*Pdx1*), and *neurogenin-3* (*NGN3*). Total RNA was isolated from homogenized pancreas samples by TRIzol reagent (Life Biolab, Hanseatic, Hamburg, Germany) according to the manufacturer’s protocol. The quantity and quality of the purified RNA were verified using a NanoDrop spectrophotometer (Thermo Scientific™). All purified RNA samples were stored at −80°C. The concentration of the extracted RNA was measured using a nanodrop device, and they were kept frozen at -80°C until cDNA synthesis.

 Moreover, 500 ng of RNA sample was used for complementary DNA (cDNA) synthesis using a cDNA synthesis kit (Thermo Scientific RevertAid First Strand cDNA synthesis kit, UK), and RT-PCR was performed. The primers of the desired genes are presented in Table 1. PCR amplification conditions comprised 15 min at 95°C, followed by 40 cycles of denaturation for 10 min, and annealing and extension at 60°C for 60 sec. Relative gene expression levels were calculated using comparative quantitative analysis (2^-ΔΔCT^) using (Gapdh) as the reference gene (Mirzapur et al. 2018). 

Thereafter, cDNA synthesis was performed using the Thermo Scientific RevertAid First Strand cDNA synthesis kit and following the manufacturer's instructions. Briefly, 500 µl of RNA along with the contents of the cDNA synthesis kit [RNA (2 µl), oligo dT (1 µl), random hexamer (1 µl), primer (15-20 pMol), 5X reaction buffer (4 µl), RiboLock RNase inhibitor (20 U/ml) (1 µl), 10 mM dNTP mix (2 µl), RevertAid M-MuLV RT (200 U/µl) (1 µl) nuclease free water (Up to 20 µl)] were transferred into a sterile and Rnase/Dnase-free microtube at 4°C (on ice), and the microtube was spun and then placed in a thermal cycler. The temperature program given to the device, according to the instructions provided in the kit, was 95°C (1 min), 42°C (60 min), and 25°C (5 min). After production, the reaction product was stored in a freezer at -80°C (Mirzapur et al. 2018). 

Polymerase chain reaction was performed using an Amplicon master mix (AMPLICON, Stenhuggervej, Denmark). The primers used were purchased from Synaclone and are listed in the table 1. Reaction materials on 2-ml sterile strips were combined and, after microspinning, were transferred to the real time machine (Step One, Applied Biosystems, USA).

### Data analysis

After ensuring the normal distribution of the data using the Kolomogrov-Smirinov test, the quantitative data was analyzed and the results were compared among groups by one-way analysis of variance (ANOVA) and Tukey post hoc tests. In all tests, p<0.05 was considered the significance level.

## Results

### Biochemical data

**Figure 1:**
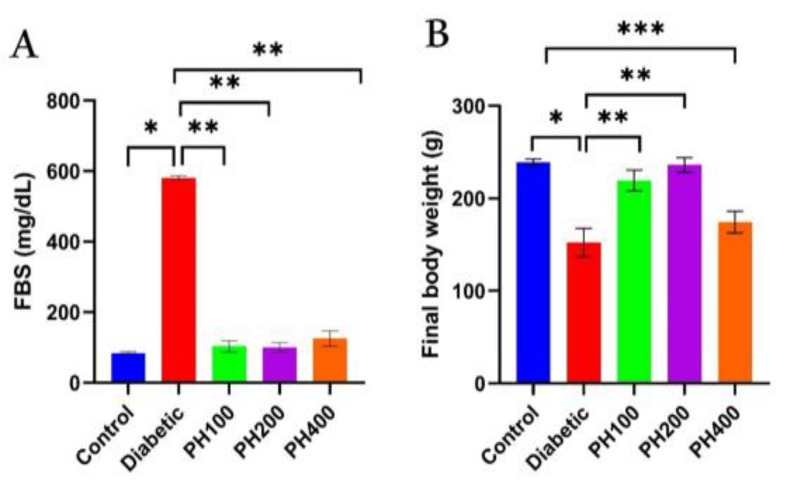
FBS (fasting blood sugar) changes (A) and final body weight of animals (B) in control, diabetic (55 mg/kg STZ) and diabetic treated with seed extract of P. harmala (PH) (PH100, PH200, and PH400 mg/kg) groups. Significant difference between groups: *p<0.05 and **p<0.01

**Figure 2:**
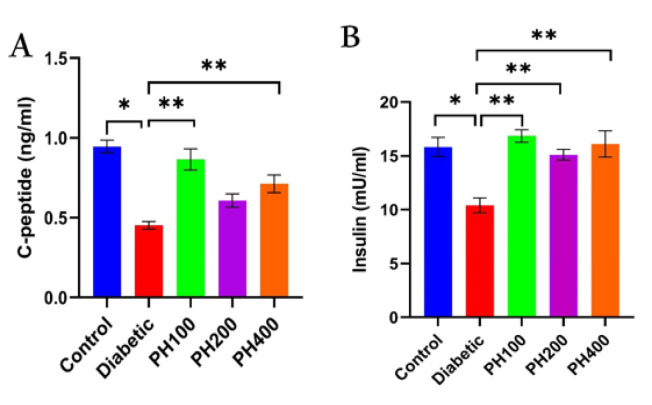
Changes in C-peptide (A) and insulin (B) in control, diabetic (55 mg/kg STZ) and diabetic treated with seed extract of P. harmala (PH) (PH100, PH200, and PH400 mg/kg) groups. Significant differences between groups: *p<0.05 and **p<0.01.

**Figure 3:**
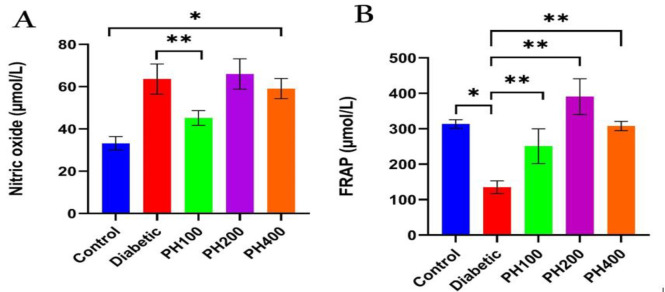
NO (nitric oxide) changes (A) and TAC (total antioxidant capacity) (B) in the control, diabetic (55 mg/kg STZ) and diabetic treated with seed extract of P. harmala (PH) (PH100, PH200, and PH400 mg/kg) groups. Significant difference between groups: *p<0.05 and **p<0.01.

Inducing T1D with STZ caused a severe increase in FBS, but treatment with *PHSE* in all three doses (100, 200, and 400 mg/kg) caused a significant decrease in FBS (p<0.01) and it reached same level as the control group (Figure 1A). After T1D induction, the weight of the animals showed a significant decrease; the treatment groups, however, showed improvement. A relative return of weight was observed in the 100- and 200-mg/kg treatment groups (Figure 1B). C-peptide and insulin levels in the diabetic group decreased significantly compared to the control (healthy) group, but increased considerably in the group treated with *PHSE* compared to the diabetic group (p<0.01) (Figure 2A and B). Diabetes caused a considerable increase in NO levels; treatment with *PHSE*, however, caused a significant reduction (p=0.002) in NO levels in *PHSE* doses (100 mg/kg) (Figure 3A). TAC showed a significant decrease in diabetic animals (p=0.001), and its relative return was observed in the treatment groups (Figure 3B).

### Gene expression

**Figure 4:**
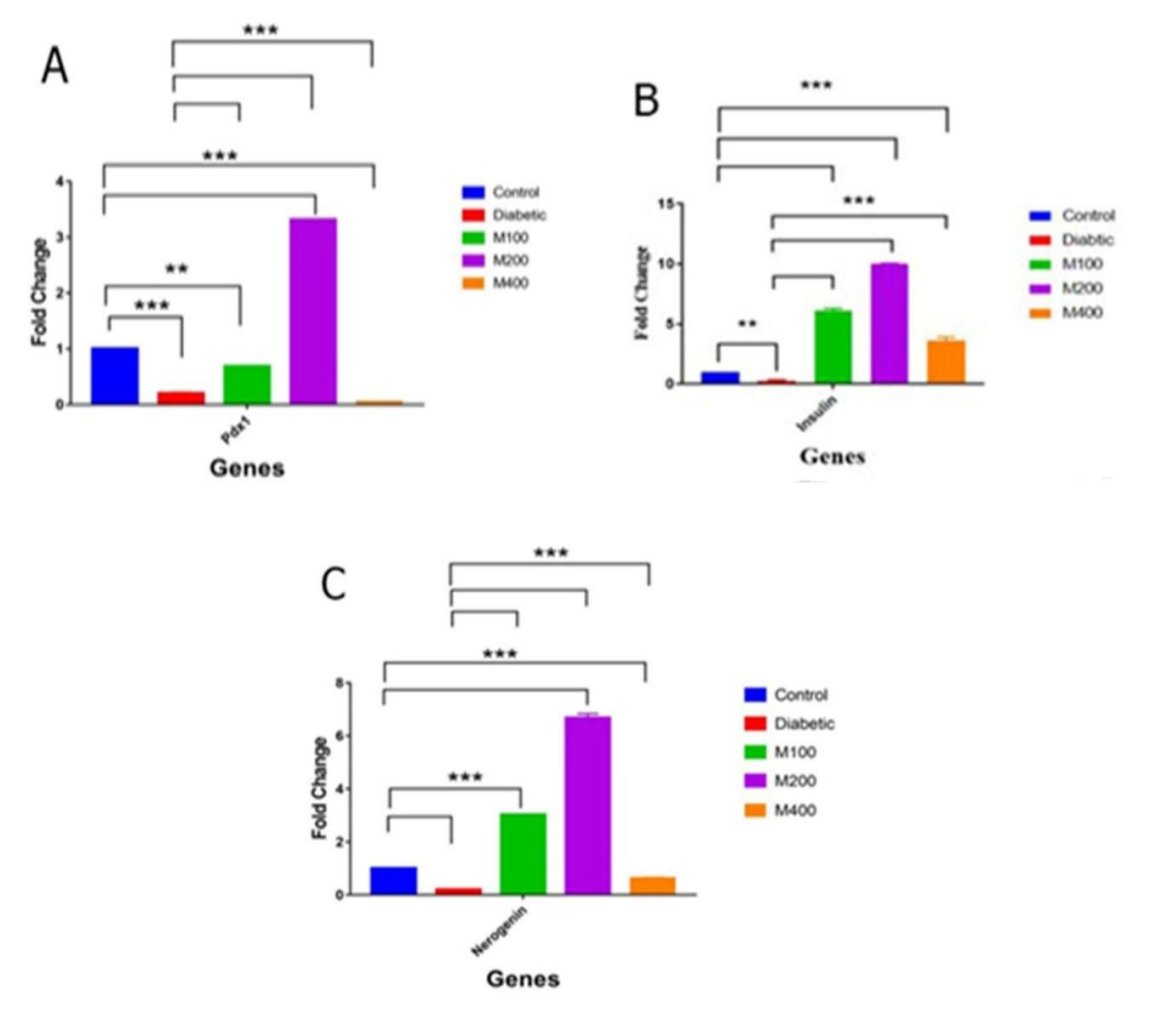
Genes expression: Pdx1 (pancreatic-duodenal gene-1) (A), Insulin (B) and NGN3 (neurogenin 3) (C): in the control (blue), diabetic (55 mg/kg STZ) (red) and diabetic treated with P. harmala seed extract: [100 (M100), 200 (M200) and 400 (M400) mg/kg]; **p<0.001and ***p<0.001.

*Pdx1* gene expression in diabetic animals was significantly decreased compared to the control group (p=0.002). In contrast, it increased in extract doses of 100 and 200 mg/kg in parameters doses had a significant difference with the diabetic group (p=0.002) (Figure 4A). *Insulin* gene expression in diabetic animals showed a significant decrease compared to the control group (p<0.01). In all extract doses, a significant increase in *insulin* gene expression was observed compared to the diabetic and even the control groups (p=0.001). Doses of 100 and 200 mg/kg of the extract showed more significant increases than the 400 mg/kg dose (Figure 4B). Similar changes were observed for the *NGN3*gene. The highest increase in the expression of this gene was observed with the 200 mg/kg dose (Figure 4C).

### Histologic evaluation

**Figure 5:**
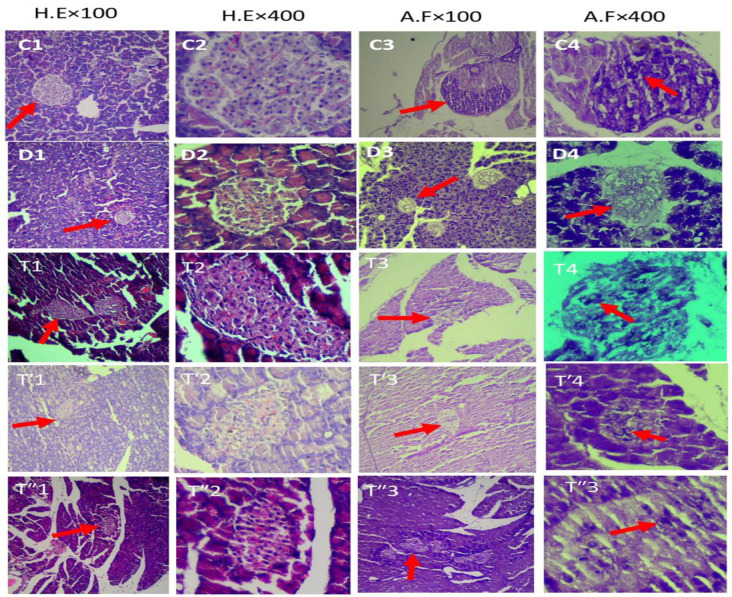
Microscopic photos from: Control (C1-4), Diabetic (D1-4), 100 mg/kg (T1-4), 200 mg/kg (T'1-4) and 400 mg/kg (T''1-4) groups with X100 (Bar: 200 µm) and X400 (Bar: 100 µm) magnification. Arrow shows islets in the first and third columns and beta cells in the fourth column.

Examining tissue sections of the pancreas with H&E and A.F staining, for better understanding of tissue changes, we used different magnifications and presented just X100 and X400 magnification photos which showed general (total) and islet cell changes respectively. Histologic evaluation revealed that diabetes caused a severe decrease in the number of islets, their cell density, beta cells, the capillary network, and the appearance of intercellular cavities. The various groups treated with *PHSE* showed improvement in the tissue structure of pancreatic islets, and a relative increase in the frequency and dimensions of islets and insulin-secreting beta cells was observed compared to the diabetic group, especially in the 200 mg/kg *PHSE* group (Figure 5).

## Discussion

In this experimental *in vivo* study, diabetic rats showed severe increases in FBS and NO levels and decreases in insulin, C-peptide, TAC, body weight, beta cell mass, and expression of related genes (*insulin, Pdx1*, and *NGN3*). In all cases, the differences between diabetic and control groups were significant. *PHSE* showed significant hypoglycemic and antidiabetic effects on the serum and pancreatic tissue of rats and caused a significant improvement in FBS (in a dose independent manner), insulin, C-peptide, TAC, body weight, number of pancreatic beta cells, and the expression of related genes. In this study, all three doses showed same significant effect on FBS and insulin, but there were differences between doses in 

improvements of the other items. It is possible to see dose dependent effect in other treatment period or lower doses. The various effects could relate to different compounds found in *PHSE*. Singh et al. showed antidiabetic effect of *P. harmala* (150 and 250 mg/kg) near to our study (Singh et al. 2008). 

In the current study, *PHSE* was observed to have considerable antidiabetic and therapeutic effects, including reduced FBS, which have been noted in some previous studies (Komeili et al. 2016; Li 2024; Singh et al. 2008), and improved serum levels of C-peptide, insulin, and antioxidant markers (TAC and NO); enhanced pancreatic specific gene expression (*insulin, Pdx1*, and *NGN3*); and an increase in beta cells, which have received less attention in previous studies.

Continuous efforts are being made to find a long-term, stable treatment for diabetes, especially type 1. In a screening of more than 100,000 drugs, only harmine stimulated the proliferation of human beta cells. A new study found that glucagon-like peptide-1 (GLP-1) receptor agonists, or harmine, increase the growth of beta cells by 5-6% (Ackeifi et al. 2020). Harmine is one of the main components of *PHSE*, and since long ago, *P. harmala* has been one of the most important medicinal plants. It contains valuable and essential alkaloids from the beta-carboline family (such as harmaline, harmine, and harman) (Asadzadeh et al. 2021).

According to histological staining (H & E and A.F) in the present study, hydroalcoholic extract of *PHSE* was associated with an increase in beta cells, especially at the dose of 200 mg/kg. Furthermore, harmine can stimulate proliferation of adult human beta cells in culture medium (Eguchi et al. 2022). Moreover, treatment with harmine caused a threefold increase in the number of beta cells, which led to improved FBS control in three groups of mice in which human diabetes was mimicked by medical engineering (Wang et al. 2015). 

In recent years, the signaling pathways and genes that stimulate the proliferation of beta cells have been identified. Similar to the present study, previous analogous studies showed that a natural compound called resveratrol causes insulin secretion and maturation of pancreatic endocrine precursors towards β-cell phenotype through *Pdx1* gene activation by way of the phosphatidylinositol 3-kinase (PI3K)/protein kinase B (AKT) signaling pathway (Pezzolla et al. 2015). In the current study, *Pdx1* gene expression and *NGN3* gene repair showed significant increases at the 200- and 100-mg doses compared to the diabetic groups and even greater increases than the control groups (non-diabetic mice). 

A particular enzyme called dual specificity tyrosine-regulated kinase-1a (DYRK1A) is probably the target of harmine. Harmine causes an increase in the amount of other known stimulators of cell division, such as c-MYC protein (He et al. 2011), by reacting with the DYRK1A enzyme. Previous studies determined that the c-MYC pathway is not a therapeutic target for beta cell regeneration, because when activated at high doses, it causes beta cell death. Nonetheless, a recent study determined that harmine increased c-MYC levels only moderately, thus not causing the death of beta cells (Dirice et al. 2016; Wang et al. 2021).

It should be noted that at high doses, beta-carboline compounds of *P. harmala* have hallucinogenic effects and are toxic for humans (Mahmoudian et al. 2002) because of the inactivation of the Monoamine oxidase (MAO) system in the liver. Additionally, while determining the toxic dose [lethal dose (LD) 50] of* P. harmala* in small mice in the current study, intraperitoneal injection of *PHSE* was severely hypoglycemic and fatal. Therefore, further investigation is required before the results of this animal study can be used in the treatment of human diabetes. 

The present study determined the beneficial effects of *PHSE* at the studied doses and in a 3-week treatment period. Additionally, two rats from each treated group were kept for one month after cessation of treatment, at which time their FBS was determined. The rats did not return to diabetic conditions, and their FBS level was normal. The experiments should be repeated with a shorter study period and lower doses to identify the effectiveness of lower doses (reduction of adverse effects of *P. harmala* consumption) and more extended maintenance of diabetic animals after termination of treatment to confirm the non-recurrence of the disease.

In conclusion, *PHSE* showed significant antidiabetic and therapeutic effects in treating and stopping T1D in rats.

**Table 1:** The primers of Insulin, PDAX1 and NGN3 genes.

Rattus norvegicus *pancreatic duodenal homeobox 1 (Pdx1*)			
F	GGTGCCAGAGTTCAGTGCTAA		
R	CCAGTCTCGGTTCCATTCG		
Product length	249		
Rattus norvegicus *neurogenin-3 (NGN3*)			
F	AAGCAGAGGAGAGCCGTAG		
R	GAACAAGAGCCAGTGAGGTAA		
Product length	149		
Rattus norvegicus *Insulin*			
F	5'-CCATCAGCAAGCAGGTCAT-3'		
R	5' TGTGTAGAAGAAACCACGTTCC-3'		
Product length	167l		
Rattus norvegicus *glyceraldehyde-3-phosphate dehydrogenase (Gapdh*),			
F	AGACAGCCGCATCTTCTTGT		
R	CTTGCCGTGGGTAGAGTCAT		
Product length	207
